# Endotyping Chronic Respiratory Diseases: T2 Inflammation in the United Airways Model

**DOI:** 10.3390/life14070899

**Published:** 2024-07-19

**Authors:** Pasquale Ambrosino, Giuseppina Marcuccio, Giuseppina Raffio, Roberto Formisano, Claudio Candia, Fabio Manzo, Germano Guerra, Ennio Lubrano, Costantino Mancusi, Mauro Maniscalco

**Affiliations:** 1 Istituti Clinici Scientifici Maugeri IRCCS, Scientific Directorate of Telese Terme Institute, 82037 Telese Terme, Italy; 2 Istituti Clinici Scientifici Maugeri IRCCS, Pulmonary Rehabilitation Unit of Telese Terme Institute, 82037 Telese Terme, Italy; giuseppina.marcuccio@icsmaugeri.it (G.M.); giuseppina.raffio@icsmaugeri.it (G.R.); 3 Istituti Clinici Scientifici Maugeri IRCCS, Cardiac Rehabilitation Unit of Telese Terme Institute, 82037 Telese Terme, Italy; roberto.formisano@icsmaugeri.it (R.F.); ennio.lubrano@unimol.it (E.L.); 4 Department of Clinical Medicine and Surgery, Federico II University, 80131 Naples, Italy; claudio.candia@unina.it; 5 Fleming Clinical Laboratory, 81020 Casapulla, Italy; fabioce26@gmail.com; 6 Department of Medicine and Health Sciences, University of Molise, 86100 Campobasso, Italy; germano.guerra@unimol.it; 7 Department of Advanced Biomedical Science, Federico II University, 80131 Naples, Italy; costantino.mancusi@unina.it

**Keywords:** chronic obstructive pulmonary disease, asthma, chronic disease, disability, exercise, rehabilitation, outcome

## Abstract

Over the past 15 years, the paradigm of viewing the upper and lower airways as a unified system has progressively shifted the approach to chronic respiratory diseases (CRDs). As the global prevalence of CRDs continues to increase, it becomes evident that acknowledging the presence of airway pathology as an integrated entity could profoundly impact healthcare resource allocation and guide the implementation of pharmacological and rehabilitation strategies. In the era of precision medicine, endotyping has emerged as another novel approach to CRDs, whereby pathologies are categorized into distinct subtypes based on specific molecular mechanisms. This has contributed to the growing acknowledgment of a group of conditions that, in both the upper and lower airways, share a common type 2 (T2) inflammatory signature. These diverse pathologies, ranging from allergic rhinitis to severe asthma, frequently coexist and share diagnostic and prognostic biomarkers, as well as therapeutic strategies targeting common molecular pathways. Thus, T2 inflammation may serve as a unifying endotypic trait for the upper and lower airways, reinforcing the practical significance of the united airways model. This review aims to summarize the literature on the role of T2 inflammation in major CRDs, emphasizing the value of common biomarkers and integrated treatment strategies targeting shared molecular mechanisms.

## 1. Introduction

With an increasing worldwide incidence and over 4.0 million annual deaths globally [1], chronic respiratory diseases (CRDs) are a leading cause of physical, psychological, and occupational disability [2,3,4], resulting in increased healthcare costs and specific rehabilitation needs [5,6,7]. Although the concept of CRDs might seem contradictory or outdated in the context of precision medicine, it remains highly relevant in terms of global health policies [8,9]. Consequently, the World Health Organization (WHO) has established the WHO Chronic Respiratory Diseases Programme to support governments in their efforts to reduce the burden of morbidity, disability, and premature mortality associated with CRDs [10]. While the term CRDs is traditionally associated with more common conditions like asthma, chronic obstructive pulmonary disease (COPD), or interstitial lung disease, the Global Burden of Disease Dictionary extends it to all pathological conditions affecting any part of the airways, from the nose to the alveoli [9]. In this scenario, the perspective of considering the upper and lower airways as a unified system has emerged as a promising and contentious topic, with the potential to revolutionize the management of CRDs [11]. Based on several shared characteristics, this model posits that the upper and lower respiratory tracts are part of a single, integrated system rather than two distinct entities [12]. Hence, it is reasonable to assume that recognizing airway pathology as an integrated entity could profoundly impact healthcare resource allocation and guide the implementation of pharmacological and rehabilitation strategies [13].

In the era of precision medicine, endotyping has emerged as another novel approach to CRDs, whereby pathologies are categorized into distinct subtypes based on specific molecular mechanisms, gene expression patterns, and immune fingerprints [14]. This approach holds the potential to refine our understanding of the biological basis of CRDs, thereby enabling precise and targeted treatment strategies tailored to the specific endotype rather than solely focusing on clinical manifestations [15]. In this regard, endotyping CRDs has contributed to the growing acknowledgment of a group of conditions that, in both the upper and lower airways, share a common type 2 (T2) inflammatory signature [16]. These phenotypically diverse pathologies, spanning from allergic rhinitis to severe asthma, may often coexist, while also sharing a number of diagnostic and prognostic biomarkers as well as therapeutic approaches tailored to common molecular pathways [17]. Therefore, T2 inflammation may represent a unifying endotypic trait of the upper and lower airways, reinforcing the practical significance of the united airways model [13].

This comprehensive review aims to summarize the literature’s findings on the role of T2 inflammation in major CRDs of the upper and lower airways, emphasizing the usefulness of common biomarkers and unified treatment strategies directed at shared molecular mechanisms.

## 2. The Unified Airways Theory

From the nose to the alveoli, the airways share most of their anatomical and functional features in both physiological and pathological conditions, thus hypothetically determining a single morpho-functional unit [18]. As a matter of fact, the perspective of viewing the upper and lower airways as a unified system is traditionally based on two fundamental observations, namely the frequent association of their pathological conditions and the evidence of joint histopathological features with common innate and adaptive immune mechanisms [12]. This concept, now widely studied as the theory of the unified airways, dates back to more than 2000 years ago when Galenus (129–201 AD) first hypothesized that asthma and chronic rhinosinusitis (CRS) were comorbidities generated by the same pathogenetic mechanism, namely “a secretion that descends from the brain through the nose into the lungs” [19]. This ancient intuition is now supported by the evidence that over 80% of patients with asthma have allergic rhinitis and about 15%-38% of patients with rhinitis have asthma, with nasal symptoms being reported in up to 85% of asthma patients [20]. Although less frequent, the co-occurrence of rhinitis and asthma appears to extend to nonallergic forms of rhinitis as well [21]. This direct association between inflammation in the nasal mucosa and the lower airways is also supported by a significant correlation between the inflammatory profiles of nasal and bronchial biopsies in patients with CRS with nasal polyps (CRSwNP) [22]. Accordingly, it has been observed that the presence of nasal polyps may be linked to a greater decline in lung function and worse clinical outcomes in asthma patients [23]. Aspirin-exacerbated respiratory disease (AERD) is another condition characterized by the involvement of both the upper and lower airways [24]. In 1968, Samter and Beers described it as a triad characterized by the presence of nasal polyps, asthma, and aspirin sensitivity [25]. Later, it was reported that the hallmark of this condition is represented by exceptionally high levels of tissue and blood eosinophils [26], thus leading to the acknowledgment that this condition is characterized by T2 inflammation along with an overproduction of cysteinyl leukotrienes and inflammatory prostaglandins [27].

Collectively, the observation that some phenotypically diverse pathologies frequently coexist has prompted a more thorough investigation into the hypothesis that they may be linked conditions manifesting in different locations, with inflammation serving as the linking factor [28]. This has even led the Global Asthma Association to take a clear position on united airway diseases, advocating for specific diagnostic and therapeutic strategies, as well as targeted education for medical students, physicians, health professionals, patients, and caregivers [29]. Translational research has further supported this hypothesis, confirming that the cascade of events resulting from mucosal insult and barrier disruption may lead in both the upper and lower airways to the production and release of specific proinflammatory cytokines with a common inflammatory signature (or endotype) [30].

## 3. Inflammatory Endotypes in CRDs: Focus on T2 Inflammation

In recent times, there has been a surge in the popularity of the endotype-driven approach to CRDs in both scientific research and clinical practice [13]. This has led to the identification of three distinct immunological and inflammatory profiles, based on the prevalent activity of different types of T-helper (Th) cells, which modulate diverse molecular pathways and cytokine production [31]. Thus, the Th1/Th2 paradigm has been updated and extended in recent years with the identification of a third subset of effector T cells, known as Th17 cells [32]. In this regard, asthma has served as a sort of prototype in endotyping CRDs, leading to the identification of two major disease categories, namely those predominantly driven by T2 inflammation and those that are not [33].

Interferon-gamma (IFN-γ) and tumor necrosis factor-alpha (TNF-α) are the main proinflammatory cytokines contributing to Th1-driven inflammation, which often results in neutrophilic infiltration with mucus hypersecretion and tissue damage, eventually leading to chronic bronchitis and emphysema [31]. Accordingly, Th1 responses are commonly observed in COPD and non-eosinophilic asthma, where neutrophilic inflammation predominates [34]. Th17 inflammation is also characterized by prevalent neutrophilic infiltration, driven by the production of cytokines such as interleukin (IL)-17 and IL-22 [35]. Mounting evidence indicates that Th17 immune response may coexist with both Th1 and Th2, thus exhibiting a closer interconnection than previously assumed [31]. For example, IFN-γ produced by Th1 cells has the capacity to stimulate the differentiation of Th17 cells, whereas IL-17 released by Th17 can in turn promote the generation of proinflammatory cytokines by Th1 cells. Moreover, contrary to the traditional belief that IL-17 only originates from Th17 cells, it is now recognized that IL-17 is also generated by IL-17-producing Th2 cells [36], with a high prevalence of these dual-function cells being detected in both the blood and bronchoalveolar lavage fluid of individuals with severe steroid-resistant asthma [37]. Collectively, the Th17 inflammatory endotype and its complex crosstalk with other inflammatory patterns are frequently associated with severe asthma and CRS without nasal polyps (CRSsNP) [31].

In allergic conditions, exposure to allergens triggers Th2-mediated (or T2) inflammation. In brief, inhaled allergens are captured by antigen-presenting cells (APCs), like dendritic cells, which promote the differentiation of naïve T cells in Th2 cells [38]. Th2 cells produce a particular set of cytokines, including IL-4, IL-5, and IL-13, driving airway eosinophilia, mucus hypersecretion through goblet cells, and parenchymal remodeling [38]. IL-4 and IL-13 stimulate B cells to switch from producing IgG to IgE, which in turn bind their receptors on mast cells and basophils and sensitize them to the allergen [39]. The key effectors of T2 response remain eosinophils, whose differentiation, activation, and recruitment from the bone marrow are orchestrated by IL-5 [40]. Eosinophils are structurally highly complex cells with a number of molecules and surface receptors, including IL-4 (IL-4Rα) and IL-5 receptors alpha (IL-5Rα) [40]. Additionally, eosinophils express surface receptors for various chemotactic agents, such as the type 3 C-C chemokine (CCR3) receptor, which binds eotaxins, as well as adhesion molecules that allow interactions with airway epithelial cells and endothelial cells [41]. The biological characteristic of eosinophils lies in their intra-cytoplasmic granules, which can be released through a fragmentary degranulation mechanism or following cytolysis [42,43]. Four cationic proteins are contained in these granules, along with growth factors such as transforming growth factor β (TGF-β), metalloproteinases (MMPs), and tissue inhibitors of metalloproteinases (TIMPs), playing significant roles in fibrosis development and parenchymal remodeling [40].

**Figure 1:**
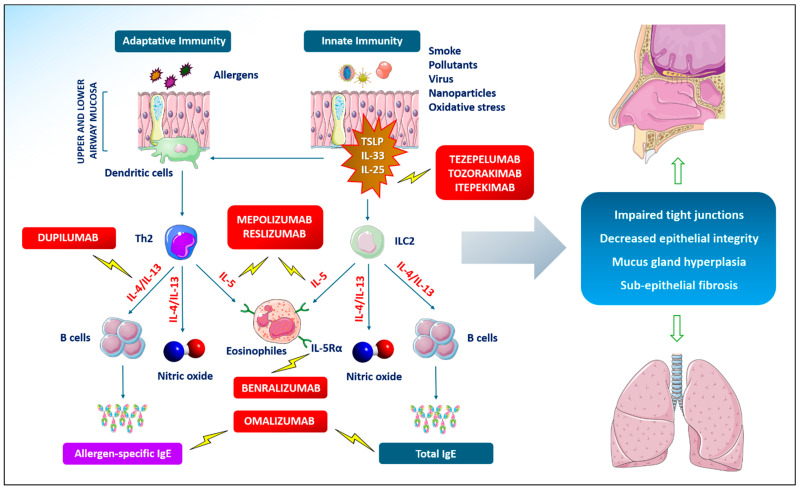
Innate and adaptive immunity in type 2 (T2) inflammation of upper and lower airways. Th2: T-helper 2 lymphocytes; IgE: immunoglobulins E; TSLP: thymic stromal lympho-proteins; IL: interleukin; ILC2s: non-specific innate lymphoid cells; IL-5Rα: interleukin-5 receptor subunit α.

While allergen-specific Th2 cells are the key effectors of adaptive immunity, non-specific innate lymphoid cells (ILC2s) also seem to be responsible for eosinophilic inflammation in CRDs characterized by a clear T2 signature even in the absence of allergen exposure [44]. The proposed hypothesis suggests that ILC2s may be activated via inflammatory mediators and alarmins released by the damaged epithelium, such as thymic stromal lympho-proteins (TSLP), interleukin (IL)-25, and IL-33 [45], which also amplify the T2 response by enhancing the ability of dendritic cells to present antigens to naïve T cells. ILC2s, in turn, release the same cytokine panel that is characteristic of Th2 responses, thus contributing to eosinophil recruitment, mucus production, and tissue repair processes seen in allergic inflammation [46] (Figure 1).

## 4. Clinical Characteristics of T2 Inflammation

Gaining a better understanding of the pathogenic mechanisms and distinct inflammatory endotypes in CRDs has led to a novel approach to CRDs, especially asthma and COPD [17]. As previously mentioned, asthma has acted as a prototype for endotyping CRDs, resulting in the identification of two primary disease categories. Therefore, these patients are now categorized as T2-high or T2-low, the former characterized by predominant eosinophilic inflammation and the latter by either neutrophilic or paucigranulocytic inflammatory response [33]. Adult asthma patients with a T2-high profile tend to report childhood onset and higher exacerbation rates [47]. Most importantly, epidemiological data suggest that most patients with difficult-to-treat asthma tend to have a T2-high disease [48]. It is also noteworthy that T2-high patients frequently exhibit specific allergen sensitization, but intermediate phenotypes of nonallergic T2-high patients also exist [49]. Conversely, these phenotypes are less contrasting for children, with no differences in lung function, exacerbations, asthma control, or steroid use when comparing T2-high and T2-low signatures [47]. The presence of an eosinophilic endotype has also been widely accepted in a relevant proportion of COPD patients, estimated to be as high as 40%, with substantial implications in terms of prognosis and therapy [50]. Data from the large Copenhagen General Population Study suggest a 1.76-fold increased risk of severe exacerbations for eosinophilic COPD patients [51]. On the other hand, based on meta-analytical data, it can be argued that eosinophilia in COPD is associated with reduced length of hospital stay and better steroid response in terms of pulmonary function [52].

In the upper airways, the role of T2 inflammation has long been recognized and even more clearly defined [53]. While there is abundant evidence for allergic rhinitis and CRSwNP [54], the clinical evidence of T2 inflammation in nonallergic rhinitis (NAR) remains more debated. NAR includes conditions with similar clinical characteristics, such as nonallergic rhinitis with eosinophilia syndrome (NARES) and nonallergic rhinitis with eosinophils and mast cells (NARESMA) [55]. These disabling diseases exhibit symptoms consistent with allergic rhinitis, but allergen skin testing shows an absence of atopy while nasal cytology reveals a characteristic eosinophilic infiltration [56]. Epidemiological data indicate that NARES and NARESMA share several clinical characteristics with other CRDs with nonallergic T2 signatures, including late-onset, association with other T2 comorbidities, and a significant response to corticosteroids [57]. Eosinophilic inflammation is also involved in most cases of CRSwNP [54], but it is less known that up to 49% of the patients with CRSsNP display a type 2 immune response as well [58]. However, compared to CRSwNP, the absolute number of infiltrating eosinophils is extensively (18-fold) lower in T2-driven CRSsNP, and this might explain why nasal polyp formation does not occur in CRSsNP despite type 2 presentation [58]. T2 inflammation, thus, appears to be implicated in most cases where mucosal damage in the upper airways results in polyp formation. Recently, it has been demonstrated that nasal polyposis in AERD also has type 2 characteristics and can be distinguished from CRSwNP by enhanced tissue eosinophilia with (surprisingly) no concomitant increases in conventional T2 inflammatory mediators associated with eosinophil proliferation and recruitment [26].

In light of the common molecular pathways and shared clinical traits, some authors have even proposed a new approach to these respiratory conditions, suggesting that labels such as CRSwNP and asthma may be oversimplified, emphasizing the identification and treatment of the common endotypic profile [17]. While this perspective is (of course) provocative and might be seen as extreme, it underscores the importance of identifying biomarkers of T2 inflammation to support personalized pharmacological and rehabilitation strategies aimed at managing recurrences, clinical deterioration, and disease exacerbation [15]. The ambitious goal is to develop unified approaches for patients with T2 inflammatory diseases of the upper and lower airways, especially when these conditions coexist [53].

## 5. Biomarkers of T2 Inflammation

Recently, strong evidence has endorsed a biomarker-driven approach to CRDs, targeting individuals with specific endotypic traits and some common clinical characteristics who are, therefore, more likely to respond to specific pharmacological and rehabilitation strategies [59]. As we discuss below, this approach has gained popularity with the growing recognition of T2-driven diseases.

### 5.1. Blood and Tissue Eosinophils

In this scenario, it becomes clear how identifying eosinophils in the bloodstream and respiratory tract fluids (such as sputum, nasal secretions, and bronchial lavage) could facilitate the recognition of T2 inflammatory patterns [60]. However, while induced sputum sampling for airway examination is the gold standard but uncommon in routine clinical practice for technical reasons [61], the count of eosinophils in peripheral blood is frequently utilized as a surrogate marker for eosinophilic airway inflammation [62]. In fact, blood eosinophils are correlated with pulmonary eosinophil counts from sputum or lung tissue in both asthmatic and COPD patients [63], although cardiac diseases in COPD and gastrointestinal reflux disease in asthma can diminish this association [64]. Collectively, it is important to highlight the evidence that this correlation is stronger in asthma than in COPD [65], with blood eosinophils representing accurate biomarkers of asthma control and airway eosinophilia across all severity degrees of asthma [66]. In this regard, one should also consider that distinct phenotypes of blood and sputum eosinophils have been observed in COPD and asthma patients, with elevated levels of CD193+ and CD66b+ eosinophils reported in COPD and increased levels of CD11b+ cells in asthma [67]. Overall, while the evidence regarding the clinical significance of eosinophils is stronger and more intuitive in the case of asthma, with a better correlation between peripheral and airway eosinophilia [68], the data on COPD are more conflicting and subject to different clinical interpretations [63]. In this regard, it has been shown that tissue eosinophils that are found in large numbers in COPD exacerbations and in smaller numbers in stable COPD are not accompanied by an increase in IL-5, suggesting that they may have different functions and recruiting mechanisms than eosinophils in asthma [69]. Accordingly, recent evidence even indicates that blood eosinophils cannot be assumed to reflect lung tissue eosinophils in COPD [70]. However, data from the large Copenhagen General Population Study cohort suggest that increased blood eosinophil levels are linked to a higher risk of exacerbations in COPD [51]. Additionally, during acute COPD exacerbations requiring hospitalization, blood eosinophilia has been shown to predict a higher short-term treatment success rate but also a higher rate of readmissions [71]. Similar conclusions on the risk of exacerbations are drawn from the SPIROMICS and the ECLIPSE studies [50,72]. However, the SPIROMICS also found that blood eosinophilia performed worse than sputum eosinophilia in identifying a subgroup of patients with reduced forced expiratory volume in 1 s (FEV_1_) and markers of emphysema on a computed tomography (CT) scan [72]. Conversely, blood eosinophilia in the ECLIPSE study was linked to higher FEV_1_, better exercise capacity, lower symptom severity and emphysema score, and an even lower bacterial load [50]. Despite some conflicting findings in the literature, it is important to highlight that peripheral blood eosinophils are, however, included in the most recent Global Initiative for Chronic Obstructive Lung Disease (GOLD) statement to drive add-on therapy with steroids in COPD [73].

Evidently, when discussing eosinophils as biomarkers of T2 inflammation, certain considerations must be made regarding the individual variability of blood and tissue eosinophils, as well as the confounding factors that can influence them. In this regard, it has been reported that a single measurement might not be sufficient in asthma due to the variability of blood eosinophils over time and evidence that roughly one in six patients whose average count is above 150 cells/μL may initially have counts below this threshold [74]. Moreover, in COPD as well as other clinical conditions, the threshold value appears to be important, with values below 150 cells/μL or above 300 cells/μL associated with a lower level of individual variability [75,76]. Numerous other factors can influence the measurement, including smoking, seasonality, technical analytical factors [75], and a range of pathological conditions such as nasal polyps, vasculitis, gastrointestinal diseases, leukemias, Hodgkin’s lymphoma, and adverse drug reactions [41]. Given that eosinophils are used to predict therapeutic responses, it is reasonable to assume that these therapies would also impact eosinophil levels. The administration of either inhaled (ICSs) or oral corticosteroids (OCSs) results in a reduction in eosinophil count in both asthma and COPD, which is dose-dependent and correlates with the effectiveness of corticosteroid treatment and suppression of eosinophilic airway inflammation [77,78]. Furthermore, in COPD, both ICSs and OCSs have been shown to significantly suppress sputum eosinophils [79,80]. When a paradoxical increase in blood eosinophiles is reported following ICS administration in COPD, more frequent exacerbations and an accelerated lung function decline should be expected [81].

**Figure 2:**
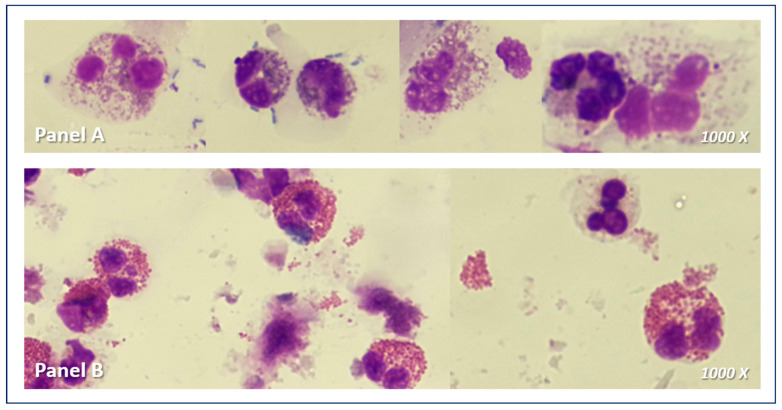
Eosinophils in nasal lavage (Panel A) and sputum cytology (Panel B).

In the upper airways, likely due to greater accessibility, the role of circulating eosinophils has been less investigated in pathological processes, with nasal cytology instead being given greater importance [82]. Increased tissue eosinophil counts have been detected in cytology following nasal curettage or in fluids obtained from nasal lavage in CRSwNP patients, showing a close association with the degree of epithelial damage and the development of polyps [82]. This aligns with the evidence suggesting that the inflammatory process driving CRSwNP is primarily mediated by eosinophils, either in the context of allergic or nonallergic rhinitis [83]. In CRSwNP, tissue eosinophilic infiltration appears to be linked with a delay in eosinophil apoptotic mechanisms, leading to an amplification of the epithelial-to-mesenchymal transition response induced by TGF-β [82,84]. A similar process occurs in the lower airways during T2-high asthma, where eosinophilic inflammation persists [85]. Accordingly, a strong correlation between nasal and sputum eosinophilia has been reported in adults with moderate-to-severe asthma, with nasal eosinophilia via quantitative cytology also linked to bronchodilator response [86]. These findings support recent research indicating similar inflammatory changes in the tissues of both asthma and rhinitis patients, reinforcing the concept of the upper and lower airways as a unified entity [87]. Therefore, assessing upper airway inflammation could offer insights into lower airway involvement, suggesting that nasal eosinophilia might serve as an alternative to sputum analysis and the nose may serve as a sort of window to the bronchial tract [87]. In this regard, due to its ease of execution and non-invasiveness, it has been reported that nasal lavage cytology can be considered a valid substitute for sputum cytology in the diagnosis of the different inflammatory phenotypes of asthma, with an even higher performance than blood cell count [88] (Figure 2).

### 5.2. Fractional Exhaled Nitric Oxide (FeNO)

Along with blood eosinophil count, fractional exhaled nitric oxide (FeNO) is widely accepted as another surrogate marker of airway eosinophilia [89]. With different functions attributed to nitric oxide (NO) in the airways (e.g., vasodilator, bronchodilator, neurotransmitter), FeNO originates from the activity of various nitric oxide synthase (NOS) enzymes, but the predominant source of elevated NO levels arises from the inducible NOS 2 (iNOS2) enzyme, expressed in epithelial and immune cells in response to inflammation [89]. In contrast to a sputum eosinophil assessment, which is technically challenging and time-consuming, FeNO is non-invasive and easily reproducible [90] and demonstrates a strong correlation with airway eosinophilic inflammation detected in induced sputum, thus offering a noninvasive means to evaluate T2 airway inflammation in asthma [91]. The correlation between blood eosinophil count and FeNO as prognostic biomarkers and their predictive role in asthma exacerbation risk has garnered interest in recent times. In a large population-based study, individuals with asthma who exhibited elevated FeNO or elevated blood eosinophiles (≥300 cells/μL) had a higher risk of exacerbation (risk ratio: 1.31), but when both biomarkers were combined, the risk ratio for acute exacerbation increased to 3.67 [92].

As a biomarker of T2 inflammation, there is a tendency for FeNO levels to correlate with increased total serum immunoglobulin E (IgE) or positive results in skin prick testing among individuals with allergic asthma [93]. Interestingly, increased FeNO lacks complete specificity for asthma, as it can also be elevated in individuals with atopy who do not display additional asthma characteristics [94]. Similarly, other clinical conditions, particularly those characterized by T2 inflammation, can affect FeNO values, including CRS and allergic rhinitis [95]. In this regard, it is important to highlight that orally exhaled gases contain NO derived from both the lower and upper airways [96]. However, although some promising results have been obtained on the use of FeNO in CRSwNP monitoring [97], its utility in upper airway diseases is limited. Nevertheless, their simultaneous presence should always be carefully considered when utilizing this biomarker in asthma.

In 2011, the American Thoracic Society (ATS) formulated a clinical practice guideline to interpret FeNO levels, defining values above 50 parts per billion (ppb) in adults and above 35 ppb in children as elevated [98]. In this regard, it is important to note that these cut-offs may vary among international guidelines and that FeNO values are influenced by various confounding factors, including age, sex, smoking habits, respiratory infections, and environmental conditions [99]. Cigarette smoking is notably significant, potentially reducing FeNO levels by up to 50% depending on the amount smoked [100]. Gender differences also play a role, with women consistently showing approximately 25% lower FeNO levels compared to men [99]. Respiratory infections, particularly those of viral origin, are linked to increased FeNO levels [99]. Additionally, supported by meta-analytical evidence, the use of ICSs and OCSs has been shown to significantly lower FeNO in both asthma and COPD patients [101,102,103]. However, numerous studies have demonstrated the ability of FeNO to predict airway inflammation, exacerbation risk, and poor asthma control [91]. Therefore, ATS guidelines suggest that FeNO levels exceeding the proposed cut-off values can indicate responsiveness to corticosteroids in asthma [98]. Recently, the potential utility of FeNO has also been proposed for monitoring T2 inflammation in clinical contexts other than asthma [91]. A post hoc analysis of the PREVENT study suggested an association between COPD exacerbations and elevations in FeNO [104], but a recent report on stable COPD patients revealed some instability in FeNO values, which seemed unrelated to eosinophil counts but somewhat associated with body weight [105]. This is partially consistent with the results of another cohort study, showing that the association between FeNO and blood eosinophils in COPD, although statically significant, is very weak [106]. Overall, this raises questions about its diagnostic and prognostic value in this clinical setting and supports the view that frequent FeNO measurements in COPD might have clinical utility in identifying individual patterns and their variations over time [107].

While FeNO is a method with numerous potential clinical applications, it cannot reveal the source of NO production. Therefore, in relatively recent times, extended NO analysis has gained traction, including measurements of NO at multiple flow rates to discern the origin of NO from alveolar or bronchial sources [108]. Partitioning exhaled NO has shown elevated distal NO levels in patients with asthma and COPD [108], with some evidence suggesting that these levels may not change with steroid treatment in either clinical settings [109]. Increased alveolar NO levels have been proposed for predicting severe, frequent, and near-term exacerbations in children with asthma [110] but not in adults with COPD [111]. In asthma, there is also emerging evidence indicating that high distal NO may be related to distal T2 inflammation and steroid-dependent asthma [112], supported by positive correlations found between alveolar NO concentration and bronchoalveolar lavage eosinophil counts [113]. In COPD, higher alveolar NO levels have been associated with poorer physical capacity and lower oxygen saturation following physical testing in stable states [114], and are linked to accelerated decline in lung function over a two-year follow-up period [115]. However, similar to FeNO, data on the clinical utility of partitioning exhaled NO as a biomarker of T2 inflammation in COPD are still debated.

### 5.3. Immunoglobulin E (IgE)

Allergen-specific IgE is integral to the pathogenesis of allergic disorders, thus representing a reliable biomarker for symptom severity in patients with allergic rhinitis [116]. Allergen-specific IgE has also demonstrated relatively high sensitivity in diagnosing asthma [117], with approximately 80% of patients with severe asthma exhibiting specific IgE levels greater than 0.35 IU/mL [118]. Conversely, the role of total serum IgE as a biomarker of T2 inflammation is less clear and remains debated. Elevated IgE levels have been observed in nonallergic late-onset asthma and nasal polyps [119], indicating that IgE-mediated disease can occur independently of allergen exposure. This is not surprising, as IL-4 and IL-13 can promote B cell class switching and IgE production [39]. Accordingly, it has been proposed that high total IgE may represent an additional biomarker for clustering T2-low asthma patients, thus identifying a group of patients with a peculiar T2-low phenotype, which is very similar to the T2-high phenotype in terms of disease severity and nasal comorbidities [120]. However, many studies have also shown that total IgE has a lower predictive value for airway eosinophilia compared to blood eosinophil count and FeNO, regardless of the cut-off used [121]. Not even this should be surprising, since IgE production occurs downstream of Th2 cell activation and the release of IL-4 and IL-13; thus, it can only address one aspect of the type 2 inflammatory response. Additionally, IgE paradoxically does not predict the likelihood of treatment response to the anti-IgE therapy with omalizumab [122]. Therefore, the primary clinical application of total IgE is to facilitate the dosing of omalizumab when initiating therapy, and its value as a biomarker of T2 inflammation should always be interpreted in combination with allergen sensitization tests, FeNO, eosinophils, and/or other emerging biomarkers [93].

Naturally, as already pointed out for other T2 inflammation biomarkers, the interpretation of specific and total IgE levels must always consider a series of modifiable and non-modifiable factors that can influence them. For example, total serum IgE concentrations are higher in males [123] and tend to increase with age [124], while allergen-specific IgE shows a clear trend toward reduction with aging [124]. Among modifiable factors, total and specific serum IgE are higher among alcohol abusers [125] and smokers [126]. Especially during exacerbations, increased total and fungus-specific IgE concentrations have been reported in fungal colonization of the airways (e.g., *A. fumigatus*) [127], including asthma with fungal sensitization (SAFS) and allergic bronchopulmonary aspergillosis (ABPA) [128]. Few results are available regarding the impact of OCS and ICS. Most seem to demonstrate that steroids can markedly reduce both specific and total IgE [129,130,131], though a temporary increase may occur after the initiation of therapy [129,132]. This should not be surprising, since serum IgE levels are known to fall when exposure to an allergen is reduced, and any treatment that reduces airway inflammation and, thus, reduces airway permeability to allergens is likely to cause a reduction in serum IgE levels [119]. However, some conflicting and contrary results have also been reported in this regard [133].

### 5.4. Emerging Biomarkers

New biomarkers for distinguishing T2 inflammation have been proposed, including the most promising serum periostin, eotaxin, and thymus and activation-regulated chemokine (TARC) [53]. The expression of periostin in the blood is triggered via the IL-4 and IL-13 via activation of the STAT6 pathway, directly leading to the transcription of the periostin gene [134]. This heightened periostin expression plays a role in tissue remodeling and fibrosis, which are crucial processes in both upper and lower airway diseases, including allergic rhinitis, CRSwNP, asthma, and COPD [13]. TARC is instead a chemokine with a high binding affinity for type 4 C-C chemokine receptor (CCR4), thus facilitating the recruitment, activation, and development of Th2 cells that express CCR4 [135]. Similarly, eotaxin selectively binds CCR3, which is expressed on both eosinophils and Th2 cells, thus controlling the migration and recruitment of eosinophiles in inflammatory sites [136].

The evidence on the potential clinical use of these biomarkers is scarce but holds promise to lay the foundation for future research. To date, sputum eotaxin concentration has been shown to be significantly raised in moderate-to-severe, but not in mild, asthma, thus suggesting that its contribution is more important in more severe disease [137]. Accordingly, elevated serum levels of eotaxin-3 have been shown to distinguish moderate-to-severe asthma from healthy individuals [138]. In allergic asthma, TARC levels are significantly higher than in healthy controls, with a negative correlation between TARC levels and FEV_1_ and a positive association with total IgE [135]. Increased blood levels of eotaxin, periostin, and TARC have also been reported in CRS as compared to healthy controls, with higher values in CRSwNP than in CRSsNP [139]. Interestingly, a decrease in these biomarkers has been observed after dupilumab therapy in CRSwNP [140]. Further studies are awaited to clarify the cost–benefit ratio in the current clinical practice of these and other promising biomarkers of T2 inflammation.

## 6. Treating T2 Inflammation: Standard Therapies and Unmet Needs

Standard treatment for respiratory diseases varies based on clinical manifestations, the location of the pathological process in the airways, its underlying mechanisms, and the presence of comorbid conditions [141]. Nevertheless, it is important to note that certain drug categories, often used in combination, are the mainstay of pharmacological treatment for different airway conditions. These include (but are not limited to) short- (SABAs) and long-acting β2 agonists (LABAs), long-acting muscarinic antagonists (LAMAs), and leukotriene receptor antagonists (LTRAs) [141]. ICSs, OCSs, and intranasal corticosteroids (INCSs), in doses ranging from low to high, are, however, the cornerstone for treating conditions of the upper and lower airways with T2 response. Accordingly, while the Global Initiative for Asthma (GINA) guidelines emphasize the use of ICS-formoterol for asthma treatment across all severity levels [142], with a high baseline FeNO predicting responsiveness [98], the GOLD guidelines recommend adding corticosteroids to dual bronchodilator therapy when eosinophil levels exceed 300 cells/µL [73]. INCS use is instead virtually recommended for all patients with CRSwNP, reserving the decision to switch to more targeted pharmacological therapies for cases that remain uncontrolled even after surgery, or for those where comorbidities with asthma or dermatological conditions necessitate an integrated approach [143].

In this regard, it has been reported that CRSwNP has a recurrence rate of up to 80%, despite medical and endoscopic surgery [144]. Similarly, other T2-driven conditions in the lower airways may remain uncontrolled with steroid-based standard treatments as well. According to the GINA guidelines, asthma control is often deemed unrealistic for a significant portion of patients due to steroid resistance and persistent uncontrolled symptoms [142]. The epidemiological data on the rate of uncontrolled asthma vary significantly depending on the geographical areas and populations considered, but the Centers for Disease Control and Prevention (CDC) report that up to 60% of adults [145] and 44% of children [146] with a confirmed diagnosis have uncontrolled asthma. Disease control may also be challenging in COPD patients, where acute exacerbations significantly impact the clinical course, causing rapid and partially irreversible lung function loss and the worsening of the long-term outcome. Frequent exacerbators, defined as those with at least two treated exacerbations per year [73], represent 22% of the COPD population and face increased hospital admissions, comorbidities, and mortality, making them a priority for research and treatment [147]. It is interesting to note that patients with higher blood eosinophil levels during stable periods tend to experience more frequent and severe exacerbations [148] and, although eosinophilic exacerbations are linked to rapid symptomatic recovery [52], this also presents an opportunity to consider eosinophilic inflammation as a treatable trait in COPD, paving the way for more personalized pharmacological and rehabilitation strategies in selected cases [53].

## 7. Precision Medicine for T2 Inflammatory Endotypes

The goal of managing various inflammatory endotypes in airway diseases is to achieve symptom control, reduce the risk of relapses and exacerbations, and prevent disease progression. In recent times, the use of biologics designed to interfere with specific inflammatory pathways has become central to this approach, thus reducing the risks of prolonged steroid therapies which include (but are not limited to) immunosuppression, weight gain, diabetes, and osteoporosis [149].

Thus, the need to selectively inhibit T2 inflammation and prevent airway remodeling induced by eosinophils has led to the introduction and commercialization of six biological therapies approved for T2-high asthma and other type 2 inflammatory diseases. These therapies encompass the anti-IgE medication omalizumab, two anti-IL-5 (mepolizumab and reslizumab), the anti-IL-5 receptor subunit α (anti-IL-5Rα) benralizumab, the anti-IL-4 receptor subunit α (anti-IL-5Rα) dupilumab, and the anti-thymic stromal lymphopoietin (TSLP) tezepelumab. Each of these therapies targets specific pathways at different levels of the T2 inflammatory cascade. Therefore, monitoring specific biomarkers of T2 inflammation may help tailor therapies to individual patients while also monitoring adherence and effectiveness [13]. In this regard, if asthma has served as a prototype disease in the testing of biologics, it is noteworthy how the GINA guidelines recommend considering their use for uncontrolled allergic asthma or for asthma with features of T2 inflammation, defined by baseline blood eosinophils ≥ 150 cells/μL, FeNO ≥ 20 ppb, or sputum eosinophils ≥ 2% [142].

### 7.1. Anti-IgE

Omalizumab is a humanized monoclonal antibody that selectively binds the high-affinity Fc epsilon receptor of IgE on eosinophils, basophils, and mast cells [150]. Based on the results of randomized controlled trials [151,152], it was first licensed in 2003 for children with uncontrolled allergic asthma aged 12 years or older, later receiving approval for pediatric use (≥6 years) due to its good safety profile in clinical trials [153]. According to GINA guidelines, sensitization to (an) inhaled allergen(s) on a skin prick testing or specific IgE should always be demonstrated before starting treatment [142]. Also used in chronic spontaneous urticaria, omalizumab has also shown effectiveness in CRSwNP with inadequate response to ICS in phase III studies [154], thus being recently approved for this indication as well. However, given its mechanism of action, omalizumab could have additional clinical applications in T2-driven diseases. This biologic has shown good efficacy and a favorable safety profile in severe asthma with overlapping COPD [155], and based on meta-analytical data from randomized controlled trials [156], also in patients with inadequately controlled allergic rhinitis. In a prospective randomized controlled open-label trial, a single injection of 300 mg of omalizumab two weeks before the start of the pollen season demonstrated better overall control of symptoms and quality of life, with the significantly reduced use of allergy symptom-relieving medications compared to standard therapies [157]. Consequently, omalizumab is also approved in Japan for treating severe Japanese cedar pollinosis, a form of seasonal allergic rhinitis that affects up to 38.8% of the Japanese population [158].

Considering that IgE production occurs downstream of Th2 cell activation and the release of IL-4 and IL-13, it can be argued that omalizumab may be effective in reducing immediate hypersensitivity reactions and acute symptoms, with limited impact on chronic inflammation and lung function [53]. For the same reason, while little to no impact on most biomarkers of T2 inflammation aside from IgE would be expected, it is noteworthy that omalizumab paradoxically increases total serum IgE by three to five times, maybe as a consequence of the prolonged half-life of IgE/anti-IgE complexes [159]. This increase in total IgE levels can persist for over a year after discontinuing treatment [160]. Paradoxically, IgE levels do not even predict the likelihood of response to omalizumab therapy [122]. Therefore, if the appropriate dose and frequency of omalizumab administration are determined by baseline IgE levels [161], this biomarker does not seem to be useful in identifying good responders and monitoring therapy. Conversely, based on the results of clinical trials such as the EXTRA study [122], the ERS/ATS guidelines for the management of severe asthma recommend using a FeNO cut-off of ≥19.5 ppb to identify patients with a higher probability of benefitting from anti-IgE treatment [162]. Contrary findings emerged from the PROSPERO study on this matter, indicating that patients responded similarly to omalizumab therapy irrespective of their initial FeNO levels [163]. Given the above conflicting results, a recent meta-analysis was performed, revealing that elevated IgE and blood eosinophil levels correlate with increased FeNO levels and that all these biomarkers of T2 inflammation may be useful for selecting asthma patients who may benefit more from omalizumab [164].

### 7.2. Anti-IL-5/5Rα

The anti-IL5 monoclonal antibodies mepolizumab and reslizumab work by preventing IL-5 from binding to its receptor, whereas the anti-IL-5Rα antibody benralizumab directly targets and blocks the α subunit of the receptor on eosinophils and basophils [53]. The results and post hoc analyses of phase III trials suggest that mepolizumab is able to enhance lung function and decrease the frequency of exacerbations in asthmatic patients with blood eosinophil levels exceeding 150 cells/µL [165,166]. In phase III trials, benralizumab also reduced annual exacerbation rates and was generally well tolerated for patients with severe uncontrolled asthma and blood eosinophils of 300 cells/μL or greater [167,168], with the phase IV SHAMAL study confirming that patients controlled on benralizumab can have meaningful reductions in ICS therapy while maintaining asthma control [169]. Similar positive results in terms of lung function and asthma control have been reported in phase III trials with reslizumab [170,171], even in the presence of CRSwNP with or without aspirin sensitivity [170]. In two randomized controlled trials, mepolizumab treatment also improved symptoms and quality of life in severe CRSwNP patients [172,173], while determining an increase in FEV_1_ after 25 weeks of treatment [172]. Recently, the results of the phase III OSTRO trial (NCT03401229) were published, showing promising outcomes also for benralizumab in reducing nasal obstruction, symptoms, and airway eosinophilia in CRSwNP. As of this review, no phase III trial on the use of reslizumab in CRSwNP has yet been concluded. Based on the available results and their mechanisms of action, major regulatory agencies have, to date, approved these biologics for treating severe eosinophilic asthma, with mepolizumab also being approved for use in CRSwNP.

In recent times, given the growing acknowledgment of the role of T2 inflammation in COPD [50], some promising findings of trials with anti-IL-5/5Rα biologics in this clinical setting are noteworthy, with further insights expected from the ongoing MATINEE (NCT04133909) and SUMMER (NCT05138250) on mepolizumab or RESOLUTE (NCT04053634) on benralizumab. A pooled analysis of the METREX and METREO trials suggests that mepolizumab reduces the annual rate of moderate/severe exacerbations by 18% compared to placebo in patients with eosinophilic COPD (≥150 cells/µL at screening or ≥300 cells/µL in the previous year) [174]. More disappointing results have been reported to date with benralizumab in phase III GALATEA (NCT02138916) and TERRANOVA (NCT02155660) studies involving COPD patients with frequent exacerbations and eosinophil counts ≥220 cells/µL [175].

An interesting aspect from the analysis of completed and ongoing trials is that, irrespective of the clinical context in which anti-IL-5/5Rα are tested, blood eosinophil counts are essential for patient selection and the prediction of therapeutic response, with a limited role of the other T2 biomarkers [165,166]. However, while IgE levels are not typically used for guiding anti-IL5/5Rα therapy, it is noteworthy that benralizumab was able to reduce total IgE levels correlated with a reduction in basophils (but not eosinophiles) and with an improvement in asthma control [176]. Some sporadic but encouraging results have also been observed regarding the potential role of FeNO in predicting the clinical response to anti-IL-5 or anti-IL-5Rα biologics. In a real-world cohort, adults with severe eosinophilic asthma who had baseline FeNO levels ≥50 ppb experienced a greater reduction in exacerbations after 12 months of these biologics compared to those with FeNO levels < 50 ppb [177]. However, in a large prospectively designed real-world trial, anti-IL-5 therapies did not appear to significantly reduce FeNO levels after 6 months of treatment, but only eosinophil levels, further confirming their role as the primary biomarker in predicting and monitoring response for these drugs [178].

### 7.3. Anti-IL-4/IL-13

Dupilumab is another monoclonal antibody that targets a component of IL-4Rα, shared by both IL-13 and IL-4 [179]. Based on the results of phase III trials in patients aged 12 years or older [180] as well as in the pediatric population [181], dupilumab is now authorized as an add-on maintenance treatment for adult and pediatric patients aged ≥6 years with moderate-to-severe asthma, characterized by an eosinophilic phenotype or steroid resistance. Interestingly, its efficacy in asthma is independent of allergic status, thus suggesting that pretreatment allergy tests may be of limited predictive value [182]. Similarly, by reducing polyp size, sinus opacification, and symptom severity, dupilumab has demonstrated efficacy and a favorable safety profile in CRSwNP [140], leading to its authorization for this condition by major medical agencies. Interestingly, when administered to patients with uncontrolled persistent asthma and comorbid perennial [183] or seasonal allergic rhinitis [184], dupilumab has shown the capacity to also reduce the rhinitis-associated symptom burden. More recently, there has also been growing interest in evaluating the use of dupilumab in eosinophilic COPD, based on encouraging results from the phase III BOREAS study (NCT03930732) conducted on COPD patients with elevated exacerbation risk despite the use of triple therapy and a blood eosinophil count of at least 300 cells/µL [185]. This multicenter study documented fewer exacerbations, less severe respiratory symptoms, and better lung function as well as quality of life in COPD patients receiving add-on dupilumab subcutaneously every 2 weeks.

In an attempt to adopt a biomarker-driven approach, the analysis of data from the phase III LIBERTY ASTHMA VOYAGE study (NCT02948959) confirmed the key role of circulating blood eosinophils and FeNO in identifying T2 inflammation and predicting the clinical response to dupilumab treatment [186]. Similar results emerged from a post hoc analysis of the phase III LIBERTY ASTHMA QUEST study (NCT02414854), which showed that an increased baseline FeNO (≥25 ppb) in asthma is associated with greater clinical effects of dupilumab versus placebo in terms of FEV_1_, independently of eosinophil levels and other clinical characteristics [187]. Another post hoc analysis of the same study confirmed the predictive value of blood eosinophils and FeNO even when employing the GINA threshold of 20 ppb [182]. Therefore, it is important to emphasize how dupilumab is the only biologic drug for which FeNO is universally accepted as a prognostic and predictive biomarker, which is independent of circulating eosinophiles and, thus, capable of guiding the therapeutic choice [187].

### 7.4. Anti-TSLP

By inhibiting TSLP, tezepelumab disrupts the T2 inflammatory cascade at an upstream point, thus reducing the production and activity of multiple inflammatory mediators. Based on the results of phase IIb PATHWAY (NCT02054130) [188] and phase III NAVIGATOR trials (NCT03347279) [189], tezepelumab is currently licensed for the treatment of severe asthma that is not adequately controlled despite high doses of ICSs plus another maintenance medication. Moreover, given its mechanism of action, this biologic drug is currently being evaluated in the phase IIa COURSE trial (NCT04039113) for use in moderate to severe COPD with frequent exacerbations despite triple therapy, and in the phase III WAYPOINT trial (NCT04851964) for its use in CRSwNP.

Similar to other biologics that target T2 inflammation, it is generally expected that tezepelumab would demonstrate greater efficacy in patients with a clear T2 signature. Accordingly, it has been shown that tezepelumab impacts all biomarkers of type 2 response, consistently reducing blood eosinophiles, FeNO, and total IgE levels in patients with moderate-to-severe uncontrolled asthma [190]. However, tezepelumab is not exclusively an anti-eosinophil drug, and it has been demonstrated that its efficacy extends beyond T2-high asthma [191]. In this regard, the results of the PATHWAY and NAVIGATOR trials show a decrease in the annualized exacerbation rate in asthma patients regardless of whether they have high or low levels of T2 inflammation biomarkers, including blood eosinophils and FeNO, although it should be recognized that patients with elevated levels of these biomarkers experience a more significant clinical improvement [191]. Accordingly, the pooled analysis of these trials suggests a reduction in exacerbation rates even in a “triple T2-low” biomarker subgroup, characterized by eosinophiles < 150 cells/μL, FeNO < 25 ppb, and no perennial allergy [191]. This should not come as much of a surprise, considering that the release of alarmins by damaged epithelium is not exclusive to T2 inflammation but rather a common defense mechanism in respiratory conditions with diverse pathogenesis [45]. Given the above, the decision to administer this medication to asthmatic patients is primarily driven by clinical criteria, specifically the inadequate response to steroid therapy, rather than the identification of elevated T2 inflammatory biomarkers.

### 7.5. Anti-IL-33/ST2

In recent times, drugs directed against IL-33 (itepekimab and tozorakimab) or its ST2 receptor (astegolimab) have held significant promise for the future of CRD treatment, with numerous trials currently registered. Among them, tozorakimab has garnered particular interest due to its dual mechanism of action, potently inhibiting IL-33 signaling on immune cells while also preventing IL-33 oxidation and subsequent epithelial dysfunction mediated by the RAGE/EGFR pathway [192]. Hence, promising yet unpublished results have emerged from the phase II FRONTIER-3 trial (NCT04570657) with tozorakimab in adult patients with moderate-to-severe asthma. In a phase II study, itepekimab has also demonstrated a lower incidence of exacerbations, while improving lung function in patients with moderate-to-severe asthma [193]. Similar results have been reported from the phase IIb ZENYATTA trial (NCT02918019), which demonstrated the efficacy of astegolimab in reducing exacerbation rates in both T2-high and T2-low uncontrolled severe asthma [194]. Some disappointing results have also emerged in patients with moderate-to-very severe COPD, where astegolimab did not significantly reduce the exacerbation rate [195]. To date, the scientific community is awaiting the results of a number of ongoing trials with anti-alarmins in COPD patients with a history of exacerbations, such as OBEORN (NCT05166889), TITANIA (NCT05158387), and its extension PROSPERO (NCT05742802) for tozorakimab, as well as the three AERIFY trials for itepekimab.

## 8. Future Perspectives

In the era of precision medicine, CRDs continue to be significant contributors to global morbidity and mortality and, despite much of this burden being preventable or treatable through pharmacological and rehabilitation strategies [196,197], these diseases still receive poor attention compared to other non-communicable diseases [9]. In this scenario, identifying common endotypes for different phenotypic manifestations is progressively paving the way for personalized treatments tailored to distinct immunological profiles of patient clusters, rather than solely addressing specific clinical manifestations [17]. The frequent coexistence of upper and lower airway diseases with a shared pathogenic mechanism is giving a further impulse to translational research on this matter, and a biomarker-driven approach is becoming crucial for the early detection of individuals who would benefit most from these targeted therapies [59]. With the growing acknowledgment of T2-driven diseases, the “one airway, one disease” paradigm is gaining traction and promises to change the future of respiratory medicine [198]. This approach is expected to not only improve patient health outcomes but also reduce future healthcare costs.

## Data Availability

Data sharing is not applicable to this article as no new data were created or analyzed in this study.
